# Antimicrobial activity and partial chemical structure of acylpolyamines isolated from the venom of the spider *Acanthoscurria natalensis*

**DOI:** 10.1590/1678-9199-JVATITD-2021-0017

**Published:** 2022-03-18

**Authors:** Tania Barth, Aline Silva, Simone Setubal dos Santos, Jane Lima Santos, Patrícia Diniz Andrade, Jessica Tsai, Eloísa Dutra Caldas, Mariana de Souza Castro, Osmindo Rodrigues Pires

**Affiliations:** 1 Laboratory of Animal Histology, Department of Biological Sciences, State University of Santa Cruz, Ilhéus, BA, Brazil.; 2 Laboratory of Microbiology, Department of Biological Sciences, State University of Santa Cruz, Ilhéus, BA, Brazil.; 3 Laboratory of Immunobiology, Department of Biological Sciences, State University of Santa Cruz, Ilhéus, BA, Brazil.; 4 Laboratory of Toxicology, Department of Pharmacy, School of Health Sciences, University of Brasilia (UnB), Brasilia, DF, Brazil.; 5 Laboratory of Toxinology, Department of Physiological Sciences, Institute of Biological Sciences, University of Brasilia (UnB), Brasilia, DF, Brazil.; 6 Laboratory of Protein Chemistry and Biochemistry, Department of Cell Biology, Institute of Biological Sciences, University of Brasilia (UnB), Brasilia, DF, Brazil.

**Keywords:** Spider venom, Acanthoscurria natalensis, Acylpolyamines, Antimicrobial, Mass spectrometry

## Abstract

**Background:**

Acylpolyamines are one of the main non-peptide compounds present in spider venom and represent a promising alternative in the search for new molecules with antimicrobial action.

**Methods:**

The venom of *Acanthoscurria natalensis* spider was fractionated by reverse-phase liquid chromatography (RP-HPLC) and the antimicrobial activity of the fractions was tested using a liquid growth inhibition assay. The main antimicrobial fraction containing acylpolyamines (ApAn) was submitted to two additional chromatographic steps and analyzed by MALDI-TOF. Fractions of interest were accumulated for ultraviolet (UV) spectroscopy and ESI-MS/MS analysis and for minimum inhibitory concentration (MIC) and hemolytic activity determination.

**Results:**

Five acylpolyamines were isolated from the venom with molecular masses between 614 Da and 756 Da, being named ApAn728, ApAn614a, ApAn614b, ApAn742 and ApAn756. The analysis of UV absorption profile of each ApAn and the fragmentation pattern obtained by ESI-MS/MS suggested the presence of a tyrosyl unit as chromophore and a terminal polyamine chain consistent with structural units PA43 or PA53. ApAn presented MIC between 128 µM and 256 µM against *Escherichia coli* and *Staphylococcus aureus*, without causing hemolysis against mouse erythrocytes.

**Conclusion:**

The antimicrobial and non-hemolytic properties of the analyzed ApAn may be relevant for their application as possible therapeutic agents and the identification of an unconventional chromophore for spider acylpolyamines suggests an even greater chemical diversity.

## Background

Compounds produced by different organisms found in nature represent a valuable alternative for the discovery of new agents with therapeutic potential [1-3]. The search for agents with antimicrobial action is especially important, given the establishment of bacterial strains resistant to conventional drugs [4]. In this context, a significant number of antimicrobial peptides (AMPs) were identified from several biological sources, including the venom of snakes, scorpions, spiders, among others [2, 5, 6]. The antimicrobial potential and information about the results of preclinical and clinical tests obtained for several of these peptides have been widely revised [1-3, 7-9]. Despite the therapeutic potential of AMPs, these molecules may have some disadvantages in terms of clinical application, including protease degradation, hemolytic action and high production cost [1, 2]. However, in addition to AMPs, spider venoms, for example, contain different biologically active molecules, including non-peptide molecules, such as organic acids, biogenic amines and acylpolyamines [10, 11]. 

Acylpolyamines are non-peptide organic molecules of low molecular weight (> 1000 Da) and represent the most abundant component of spider venom [12]. The combination of techniques, such as mass spectrometry and nuclear magnetic resonance, are important for the characterization of the chemical structure of acylpolyamines [13]. Currently, the structure of 409 acylpolyamines is available in the *venoMS* database [14]. The general chemical structure of these molecules comprises four segments, being a lipophilic aromatic acyl head, a linker amino acid residue, the polyamine backbone chain and the backbone tail [15]. Due to the possible combinations between these segments, acylpolyamines can be structurally very diverse, varying in length, number of amide bonds and functional groups [11, 16]. 

Acylpolyamines are recognized for their neuromodulatory activity on the nervous system of vertebrates and invertebrates, mainly antagonizing glutamate receptors and selectively blocking cationic channels [12, 13]. However, some studies have shown that acylpolyamines may have other biological activities, such as antimicrobial action. Antimicrobial properties have been identified in spider acylpolyamines since 2007, when Pereira *et al*. [17] identified an acylpolyamine named mygalin, isolated from the hemocytes of the spider *Acanthoscurria gomesiana*. Subsequently, acylpolyamines with antimicrobial activity were identified in the venom of *Brachypelma smithi* [18], *Nephilengys cruentata* [19] and *Vitalius dubius* [20], being only the acylpolyamine of *V. dubius*, called VdTX-I, fully described in the literature and, in none of these studies, the mechanism of antimicrobial action of acylpolyamines was investigated. 

Recently, a new study has shown that the mechanism of antimicrobial action of a synthetic version of mygalin on *Escherichia coli* involves disruption of the bacterial membrane, inhibition of DNA synthesis, the generation of reactive oxygen species (ROS), among others actions [21]. Regarding the tarantula spider *Acanthoscurria natalensis*, the present study represents the first report on the presence of acylpolyamines in the venom of this species. Thus, we present here the partial chemical structure of five acylpolyamines isolated from the venom of *A. natalensis* and the antimicrobial and hemolytic activity of these molecules.

## Methods

### Spiders and venom extraction

Female spiders of *A. natalensis* (n = 30) were collected (SISBIO license number 51803-1) from Fazenda Nossa Senhora Aparecida (GO, Brazil). The venom (~ 30 μL/animal) was extracted by electrostimulation (75 V for 3 s) between 1 and 2 times for each animal [22] (SISGEN license number A826A3A). The samples were lyophilized and stored at -20 °C until use.

### Acylpolyamine purification by reversed-phase liquid chromatography (RP-HPLC)

Acylpoliamine purification was obtained using three chromatographic steps (step 1 a 3). For the first step (step 1), the crude venom was solubilized (20 mg/mL) in solvent A [0.12% trifluoroacetic acid (TFA) (v/v) in water] and centrifuged (10,000 rpm). Aliquots of 200 μL of the supernatant were applied to a C_18_ reversed-phase column (Vydac 218TP54, 4.5 mm x 250 mm, 5 μm), previously equilibrated with the same solvent, using a flow rate of 1 mL/min. The elution of fractions was obtained using a linear gradient of 0 to 60% of solvent B [0.12% TFA (v/v) in acetonitrile (ACN)] in 60 min. The chromatographic fractions were tested for antimicrobial activity and the main active fraction, named ApAn, was rechromatographed (step 2) using solvents A [0.24% TFA (v/v) in water] and B [0.24% TFA (v/v) in methanol]. Samples of the ApAn fraction were solubilized in solvent A and centrifuged (10,000 rpm). Aliquots of 200 μL of the supernatant were applied to a Phenyl Hexyl C_18_ reversed-phase column (Phenomenex, 2.10 mm x 300 mm, 2.6 µm) previously equilibrated with the same solvent, using a flow rate of 1 mL/min. The elution of fractions was obtained using a gradient from 0 to 18% B in 40 min., 18% B from 40 to 50 min. and from 18 to 30% B from 50 to 60 min. These fractions were also tested for antimicrobial activity and those with more activity and better chromatographic resolution (named ApAn1 to ApAn5) were selected for the next rechromatography (step 3), performed as in step 2, but with a new elution gradient. In step 3, for fractions ApAn1 to ApAn3, the gradient was 0 to 20% B in 10 min. and 20% B from 10 to 40 min and for fractions ApAn4 and ApAn5, the gradient was 0 to 30% B in 10 min. and 30% B from 10 to 40 min. The main peak of each fraction obtained in this step was analyzed by MALDI-TOF to verify its molecular mass and sample homogeneity. Samples were then accumulated for analysis by UV spectroscopy and ESI-MS/MS and for determination of minimum inhibitory concentration (MIC) and hemolytic activity. The eluted fractions in each step were detected simultaneously at 216 nm and 280 nm, manually collected, lyophilized and stored at -20^o^C until use.

### MALDI-TOF/TOF

The ApAn1 to ApAn5 fractions were analyzed using a SCIEX TOF/TOF^TM^ 5800 MALDI mass spectrometer (AB SCIEX, Framingham, MA, USA) with α-cyano-4-hydroxycinnamic acid (HCCA) as the matrix. The ions were detected in reflector positive mode from m/z 500 to 2000 and the mass spectra were converted to the “.mzxml” format for data analysis using the mMass 5.5.0 software. According to the observed molecular masses, the fractions ApAn1, ApAn2, ApAn3, ApAn4 and ApAn5, were named ApAn728, Ap614a, ApAn614b, ApAn742 and ApAn756, respectively.

### Ultraviolet (UV) spectroscopy

UV analyzes were performed on UV-Visible spectrophotometer (UV-1800, Shimadzu) and the UV spectrum (200-400 nm) of ApAn728, Ap614a, ApAn614b, ApAn742 and ApAn756 solubilized in Milli-Q water was acquired at room temperature. For comparison, the UV spectrum of L-tyrosine, 5-hydroxytryptamine, histamine, L-tryptophan and L-phenylalanine compounds were also acquired under the same conditions.

### ESI-MS

ESI-MS and MS/MS analysis were performed using a 4000 Qtrap triple-quadrupole mass spectrometer (SCIEX, Framingham, MA, USA) fitted with a Turbo Ion Spray electrospray ionization (ESI) source. System operation and data acquisition were controlled by Analyst^®^ (V 1.5.1) software (SCIEX). Samples of ApAn728, Ap614a, ApAn614b, ApAn742 and ApAn756 (solubilized in methanol/water, containing 0.1% formic acid) were analyzed by direct infusion into the mass spectrometer under flow of 10 μL/min. ESI-MS/MS was performed in positive ionization mode, with multiple reaction monitoring (MRM). declustering potential (DP), collision energy (CE) and collision cell exit potential (CXP) were optimized for the three most abundant transitions for each analyte. The parameters of the mass spectrometer ion source were: entrance potential at 10 V, ion source at 500 ^o^C, ion source gas 1 (GS1) at 12 psi, ion spray voltage at 5500 V, curtain gas at 10 psi, and collision gas at medium.

### Antimicrobial activity

Antimicrobial activity of chromatographic samples was evaluated by liquid growth inhibition assays [23] against Gram-negative *Escherichia coli* ATCC 25922 and Gram-positive *Staphylococcus aureus* ATCC 25923 bacteria grown in Mueller-Hinton (MH) medium at 37 °C under agitation. After 24 h and optical density reached 1 to 590 nm, aliquots of 50 μL of each culture diluted 1:100 (*S. aureus*) and 1:50 (*E. coli*) were incubated with 50 μL of chromatographic samples (in duplicate) diluted in Milli-Q water for 24 h at 37 °C. Milli-Q water or 0.4% (v/v) formaldehyde were used as positive and negative control, respectively. Growth inhibition was determined by measuring absorbance at 595 nm in a Multiskan FC plate reader (Thermo Scientific).

### Minimum inhibitory concentration (MIC)

MIC was determined by liquid growth inhibition assays as described above (in the “Antimicrobial activity” section). Aliquots of 50 μL of the samples serially diluted from 256 µM to 4 µM for ApAn728, Ap614a, ApAn614b and 128 µM to 4 µM for ApAn742 and ApAn756, were incubated for 24 h at 37 °C with 50 μL of the *S. aureus* and *E. coli* dilution. MIC was defined as the lowest concentration that causes 100% inhibition of the bacterial growth, obtained from three or two independent experiments performed in duplicate or triplicate, according to the amount of sample available.

### Hemolytic activity

The hemolytic activity of ApAn728, Ap614a, ApAn614b, ApAn742 and ApAn756 was tested against erythrocytes of SWISS mice [24] (approved by the Ethics Committee on Animal Use, of the University of Brasilia (CEUA-UnB), under protocol UnBDoc number 44/2017). Erythrocytes were washed with Krebs solution (113 mM NaCl, 1.2 mM KH_2_PO_4_, 4.0 mM KCl, 1.2 mM MgSO_4_, 2.5 mM CaCl_2_, 25 mM NaHCO_3_, 11.1 mM C_6_H_12_O_6_, pH 7. 4) to obtain a 4% erythrocytes suspension. Aliquots of 50 µL of this suspension were incubated with 50 µL of a serial dilution (256 µM to 0.125 µM) of ApAn728, Ap614a, ApAn614b, ApAn742 and ApAn756 in a 96-well plate for 2 h at 37 ^o^C. After, samples were centrifuged (1000 x *g* for 3 min) and the absorbance of the supernatant was measured at 550 nm on Multiskan FC (Thermo Scientific). Erythrocytes incubated with Krebs solution or 1% TritonX-100 were used as a negative and positive controls, respectively. Hemolytic activity was expressed as a percentage of the positive control (100% hemolysis) from three independent experiments performed in duplicate.

## Results

### Acylpolyamine purification

**Figure 1.**
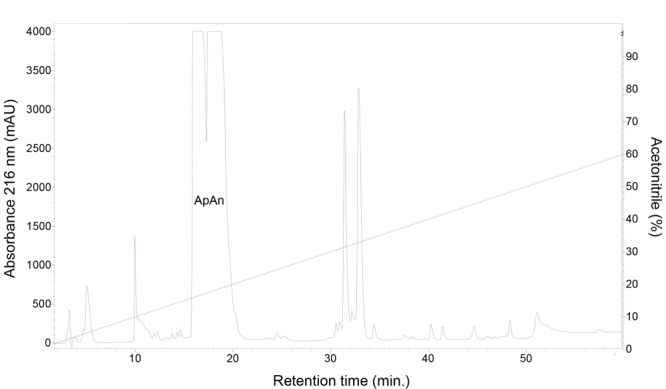
Chromatographic profile (step 1) of the total venom (4 mg) of *A. natalensis* fractionated by RP-HPLC on a column C18, under linear gradient from 0 to 60% solvent B (0.12% v/v TFA in ACN) in 60 min and flow 1.0 mL/min. ApAn: fraction of interest containing acylpolyamines.

**Figure 2.**
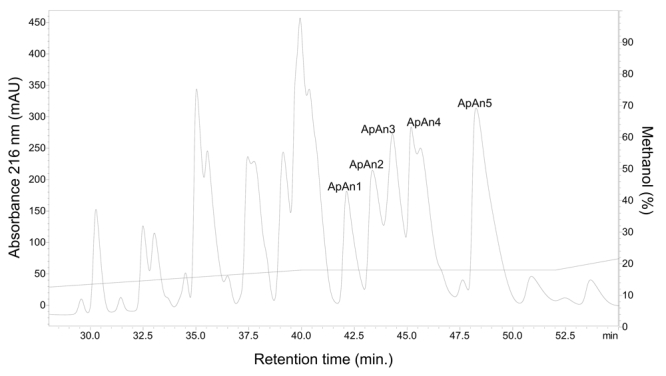
Rechromatography (step 2) of the ApAn fraction by RP-HPLC on a C18 column with optimized methodology (0 to 18% solvent B in 40 min, 18% B from 40 to 50 min and 18 to 30% B of 50 to 60 min). Solvent B: 0.24% TFA v/v in methanol. Fractions of interest were named as ApAn1, ApAn2, ApAn3, ApAn4 and ApAn5.

**Figure 3.**
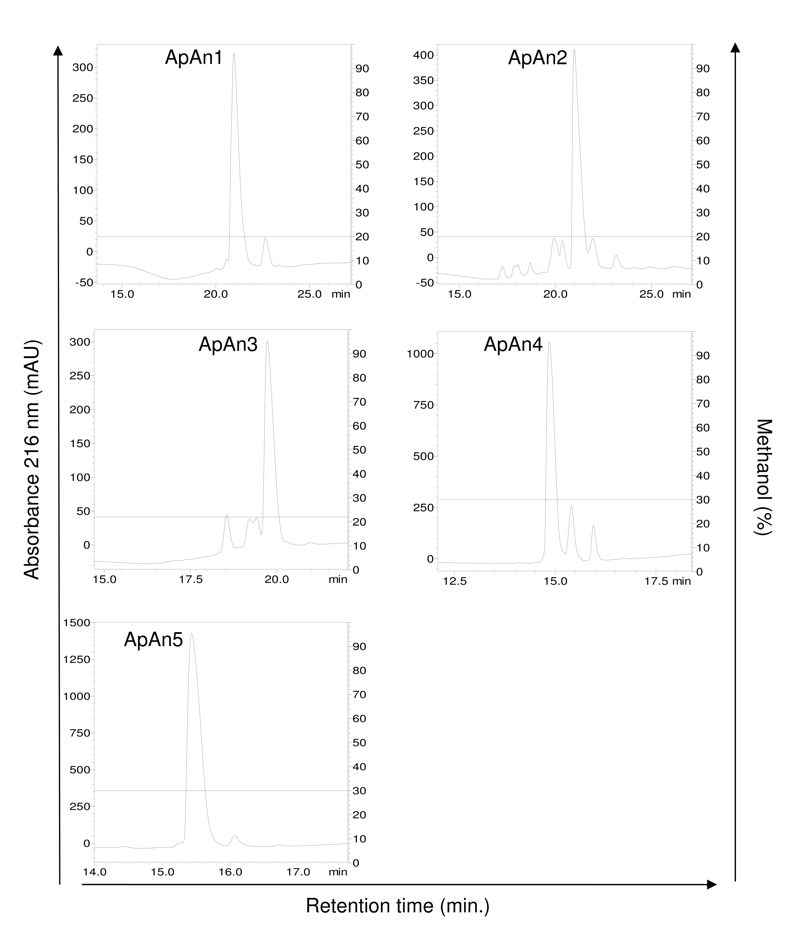
Rechromatography (step 3) of ApAn1, ApAn2, ApAn3, ApAn4 and ApAn5 fractions by RP-HPLC on a C18 column with optimized methodology (for ApAn1 to ApAn3: linear gradient from 0 to 20% solvent B in 10 min and 20% B of 10 to 40 min; for ApAn4 and ApAn5: linear gradient from 0 to 30% B in 10 min and 30% B from 10 to 40 min). Solvent B: 0.24% TFA v/v in methanol.

**Figure 4.**
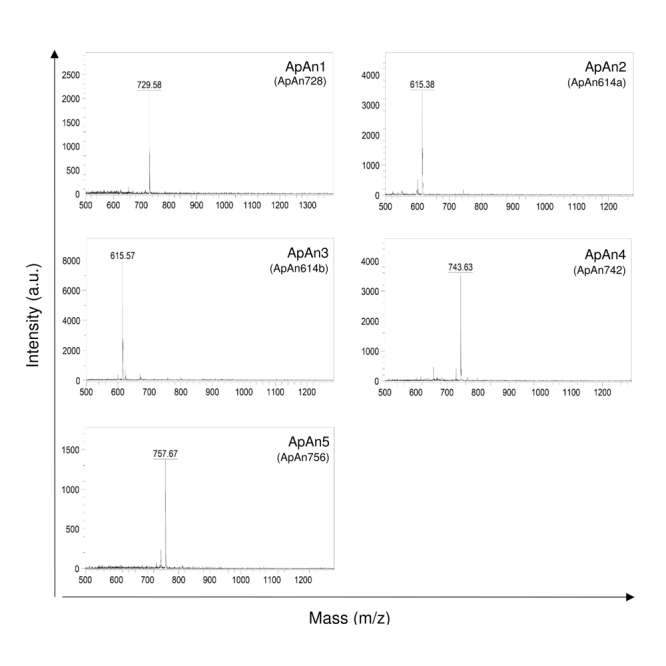
MALDI-TOF/MS spectrum from ApAn1, ApAn2, ApAn3, ApAn4 and ApAn5 (isolated in step 3) obtained in positive reflector mode. Samples in α-cyano-4-hydroxycinnamic matrix. In parentheses, nomenclature assumed according to the observed molecular mass.

The crude venom of *A. natalensis* was fractionated by RP-HPLC (step 1) (Fig. 1) and chromatographic fractions were tested for antimicrobial activity. The main active fraction, eluting between 12 and 22 min and containing the acylpoliamines, was named ApAn. Due to the large number of components, this fraction was rechromatographed (step 2) and the resulting fractions (Fig. 2) were tested for antimicrobial activity. The five major active fractions (ApAn1 to ApAn5) (Fig. 2) were selected for rechromatography (step 3) (Fig. 3), resulting in the elution of a major peak for each fraction. These peaks were analyzed by MALDI-TOF (Fig. 4) and only one major protonated ion [M+H]^+^ for each peak was identified, suggesting sample homogeneity. These ions were identified at m/z 729 (ApAn1), m/z 615 (ApAn2), m/z 615 (ApAn3), m/z 743 (ApAn4) and m/z 757 (ApAn5) and according to the corresponding molecular mass, they were finally named ApAn728, ApAn614a, ApAn614b, ApAn742 and ApAn756, respectively (Fig. 4).

### Partial characterization of ApAn chemical structure

**Figure 5.**
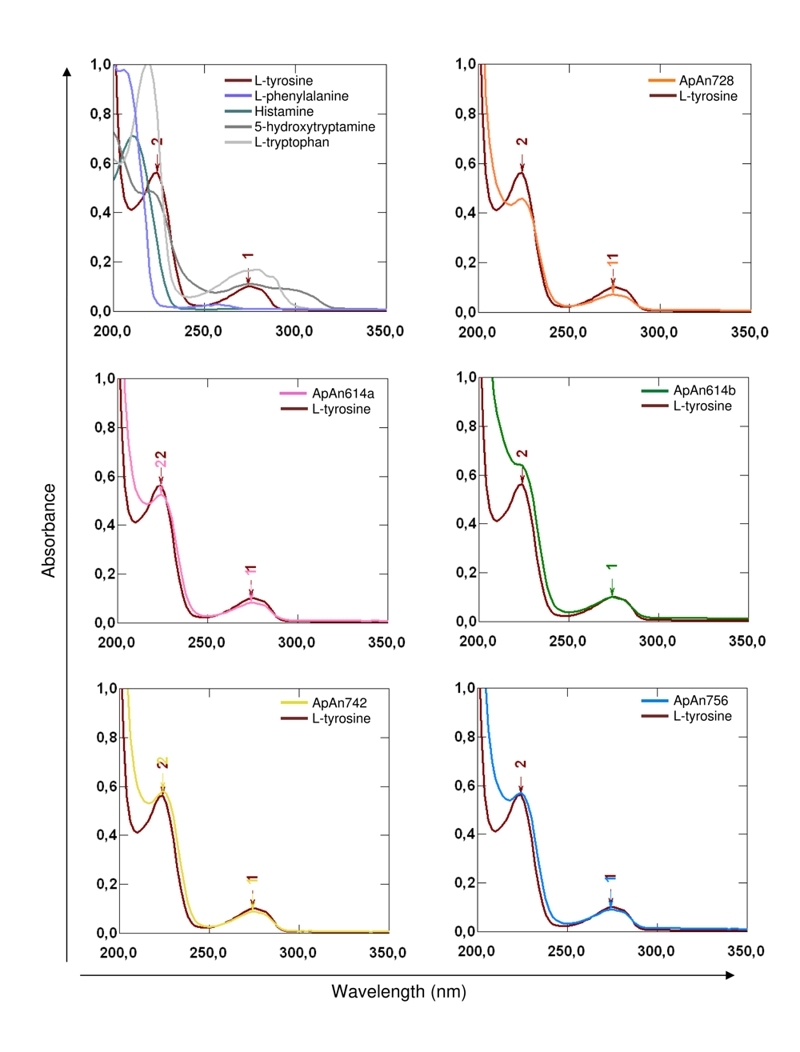
UV absorption spectrum from ApAn728, ApAn614a, ApAn614b, ApAn742 and ApAn756 compared to different compounds. The maximum values of absorption at (2) 224 nm and (1) 274 nm of the samples were coincident with the values shown by L-tyrosine. The spectra were acquired between 200 and 400 nm at room temperature.

The partial chemical structure of ApAn728, Ap614a, ApAn614b, ApAn742 and ApAn756 was suggested from the analysis of UV absorption spectra and interpretation of the fragmentation pattern obtained by the ESI-MS/MS spectra, compared with data described in the literature and database *venoMS* (https://www.venoms.ch/). Overlapping of the ApAn UV spectra with the histamine, L-tryptophan, L-phenylalanine, 5-hydroxytryptamine and L-tyrosine spectra, showed that ApAn presented the same tyrosine absorption profile, with maximum values at 224 nm and 274 nm. (Fig. 5), suggesting that the aromatic acyl group of ApAn contains a tyrosyl unit.

**Figure 6.**
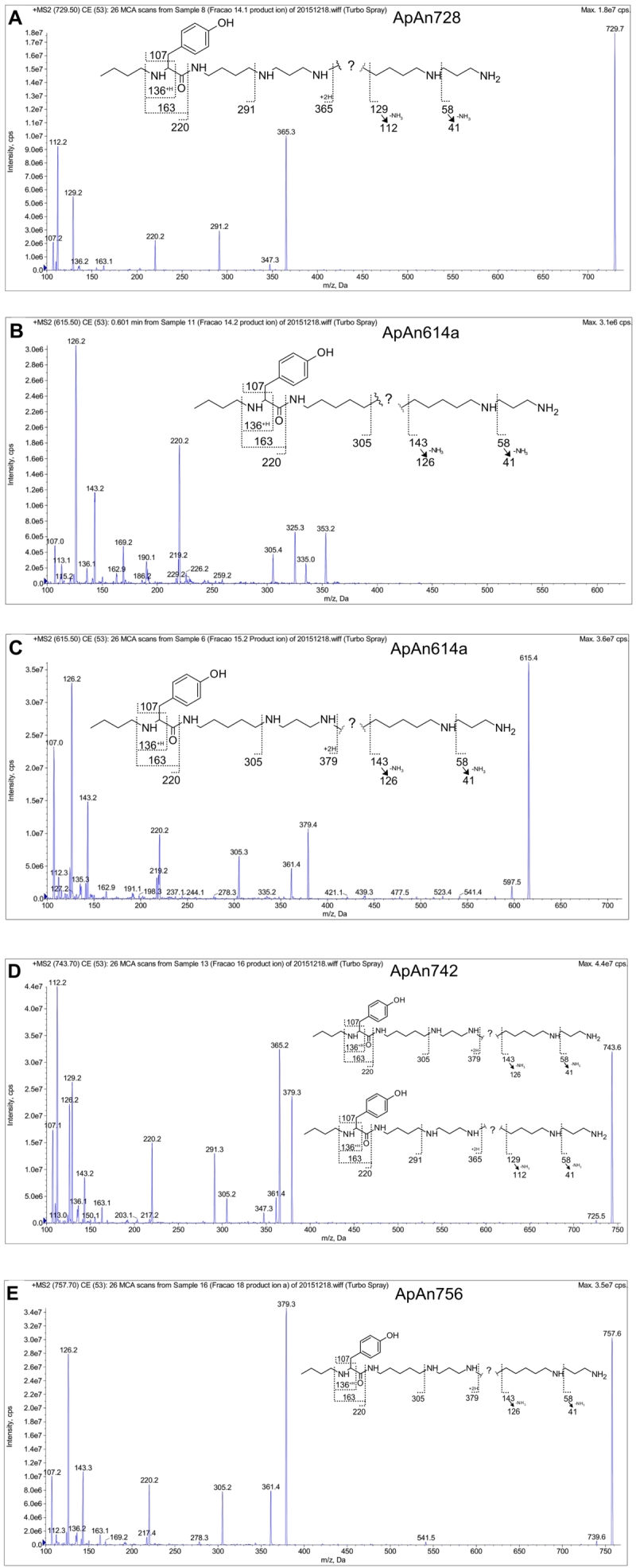
ESI-MS/MS spectra of the protonated ion [M+H]+ of **(A)** ApAn728, **(B)** ApAn614a, **(C)** ApAn614b, **(D)** ApAn742 and **(E)** ApAn756 and suggested partial chemical structure (inset). For **(D)** ApAn742, two structures were suggested due to possible co-elution of isomers in the sample.

The analysis of ApAn ESI-MS spectra showed the presence of ions at m/z 729, 615, 743 and 757 in the form [M+H]^+^ (ESI-MS and other ESI-MS/MS spectra can be found in Additional files 1-5), corresponding to the ions obtained by MALDI-TOF (Fig. 4). The MS/MS spectrum of all ApAn (Fig. 6) showed the presence of ions at m/z 163, m/z 136 and m/z 107, which were interpreted as products of the fragmentation of the tyrosyl unit, corroborating the results obtained by UV spectra. In addition, the ion at m/z 220 suggested that the tyrosyl unit is linked to butylamine group and that all ApAn have the same chemical structure in this region of the molecule.

For the initial portion of the ApAn polyamine chain, ions were observed at m/z 291 and 365 (ApAn728 and ApAn742) and at m/z 305 and 379 (ApAn614a, ApAn614b, ApAn742 and ApAn756), suggesting that the latter ions occur due to the presence of an additional methylene unit. The main ions observed above m/z 365 and m/z 379 in ApAn spectra, were related to the fragmentation products of the intermediate portion of the polyamine chain. However, these ions were not structurally indicated, as no compatible structures were found.

Regarding the terminal polyamine chain, the presence of ion pairs at m/z 129/112 (ApAn728, ApAn742) and/or m/z 143/126 (ApAn614a, ApAn614b, ApAn742, ApAn756) in the ApAn spectra, indicated the fragmentation of this portion of the polyamine chain and the loss of an NH_3_ residue, considering that the difference in mass between each pair is 17 Da. These ion pairs suggest that the structure of the terminal polyamine portion is consistent with the PA43 (129/112) or PA53 (143/126) units. In the MS/MS spectrum of ApAn742, the ions at m/z 291 and 365 and at m/z 305 and 379 (for the initial polyamine chain) were observed, as well as the ion pairs 129/112 and 143/126 (for the terminal polyamine chain), leading us to consider the possible co-elution of isomeric molecules in this sample.

Based on the above considerations, Figure 6 (inset) shows the suggested partial chemical structure for the chromophore and for the initial and terminal polyamine chain. For ApAn742, two structures have been suggested, but other options can also be considered.

### Minimum inhibitory concentration (MIC)

**Figure 7.**
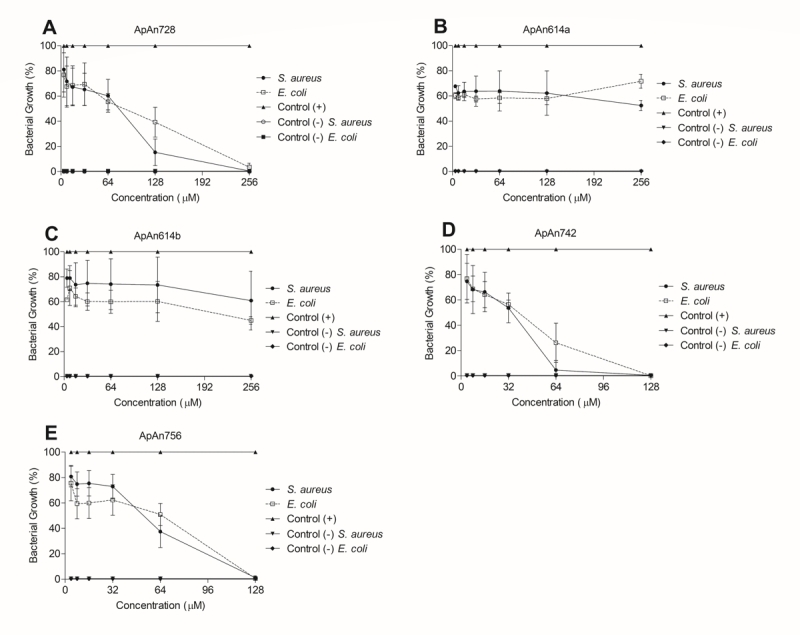
Antimicrobial activity of **(A)** ApAn728, **(B)** ApAn614a, **(C)** ApAn614b, **(D)** ApAn742 and **(E)** ApAn756 against *Staphylococcus aureus* (SA) and *Escherichia coli* (EC), as a result of the serial dilution from 256 µM to 4 µM or 128 µM to 4 µM concentration, performed to determine the MIC. Data were expressed as mean ± SD.

The antimicrobial activity of ApAn was tested against *E. coli* and *S. aureus* at different concentrations (Fig. 7). ApAn614a and ApAn614b showed low activity, inhibiting around 20-40% of the growth of both bacteria, even at the highest concentration tested (256 µM). The antimicrobial activity of ApAn728, ApAn742 and ApAn756 was dose dependent and the MIC of ApAn728 was 256 µM against *S. aureus*, while the MIC of ApAn742 and ApAn756 was 128 µM against *S. aureus* and *E. coli*.

### Hemolytic activity

**Figure 8.**
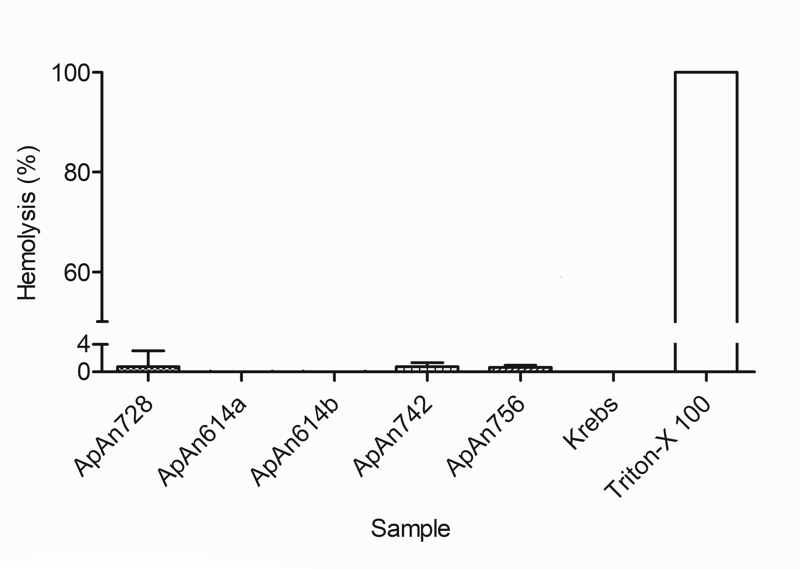
Hemolytic activity of ApAn728, ApAn614a, ApAn614b, ApAn742 and ApAn756. Krebs: buffer used as a 0% hemolysis control. Triton-X 100: used as a 100% hemolysis control. The graph represents the results obtained for the highest concentration used (256 µM). Data were expressed as mean ± SD.

The effect of ApAn728, ApAn614a, ApAn614b, ApAn742 and ApAn756 on mice erythrocytes was evaluated at different concentrations (256 µM to 0.125 µM) and until the concentration of 256 µM (shown in Fig. 8), the hemolytic activity remained around 1% only.

## Discussion

Acylpolyamines represent one of the most abundant components of the spider venom [25], reaching up to 50 different molecules in a single sample [26]. In the venom of *A. natalensis*, such abundance was also clearly verified by the chromatographic profile, where the elution of ApAn fraction occurred for approximately 4 minutes and the rechromatography of this resulted in the elution of approximately 15 fractions not fully resolved, in addition to ApAn728, ApAn614a, ApAn614b, ApAn742 and ApAn756. Molecular masses between 350 and 1000 Da have been reported for acylpolyamines isolated from spider venom [25]. ApAn showed molecular masses of 614, 728, 742 and 756 Da. Some acylpolyamines with molecular masses similar to ApAn have already been reported for other spider species. The acylpolyamine called VdTX-I, with 728 Da, was isolated from the venom of *V. dubius*, but its chemical structure has not been determined [27]. In *Aphonopelma chalcodes* two acylpolyamines were identified, with 600 Da (Apc600) and 728 Da (Apc728), being their chemical structure partially characterized [28].

The chemical structure of acylpolyamines can be characterized initially by the identification of its chromophore, through the interpretation of the mass spectrum and UV absorption pattern. The latter can be correlated with characteristic chromophores, such as for example: λ_max_ at 268, 284, and 292 nm (4-hydroxyindole) and λ_max_ at 280, 288; shoulder at λ = 270 nm (indole) [29]. These patterns have been identified, for example, in acylpolyamines isolated from the *Argiope lobata* and *Nephilengys borobonica* spiders [30, 31]. However, the UV spectrum of ApAn showed a tyrosine-like pattern with maximum absorption at 224 and 274 nm, suggesting that the chromophore of ApAn is represented by a tyrosil unit. Although this pattern is unusual, the acylpolyamines of the spider *Aphonopelma californicum* [32] and the wasp *Philanthus triangulum*, named Philantotoxins PTX-433, PTX-334 and PTX-343 [33], also have a tyrosil unit in its composition. Já a Apc600 and Apc728 from *A. chalcodes* showed a tyramine-like chromophore [28]. Furthermore, based on the analysis of the acylpolyamine mass spectra, the presence of characteristic product ions at m/z 107, 123, 130 and 146, correlated with the phenol, di-hydroxybenzene, indole and mono-hydroxyindole chromophores, respectively [26, 31], can contribute to the identification of the chromophore. Among these, only the m/z 107 ion was identified in the ApAn spectra, possibly originating from phenolic ring present in tyrosine. The m/z 136 ion, observed in different ApAn spectra, also indicates the presence of a tyrosine-like chromophore, as observed in the spectra of Philantotoxins PTX-433, PTX-334 and PTX-343 [33]. The fragmentation of the polyamine chain, in turn, results in the formation of other characteristic ions.

In the ApAn, the ion pairs m/z 129/112 and/or 143/126 were identified. These ions were related to fragmentation of the terminal portion of the polyamine chain with the neutral loss of an ammonia (NH_3_), as observed in *N. clavata* acylpolyamine JSTX-3 [34] and/or *Agelenopsis aperta* [35, 36]. These ions also suggested that the terminal portion of the polyamine chain of ApAn is formed by PA43 or PA53 units, which have already been described in previous studies [29, 37]. In addition, a possible co-elution of isomers in the sample of ApAn742 was considered, since ion pairs 129/112 and 143/126, identified in the spectra of ApAn742, cannot be present in the chemical structure of the same molecule, considering that N-atoms are separated by three to five methylene units [38]. The polyamine chain is a common component among acylpolyamines and its composition and extent can vary considerably [25], due to possible methylation and hydroxylation sites and the variable number of nitrogen atoms [38]. ApAn728, 742 and 756 have a 14 Da mass difference between them, which could be attributed to the addition of a methylene unit (CH_2_), similarly to that observed between the NPTX-943 and NPTX-957 acylpololyamines [31]. However, as it was not possible to identify the complete chemical structure of ApAn, this information could not be confirmed, as well as the difference of 114 Da of ApAn614a and ApAn614b compared with ApAn728.

Although acylpolyamines are little explored about their antimicrobial potential, they are promising, as they have other desirable characteristics for the formulation of therapeutic agents, such as the small size and the ease of obtaining synthetic analogs [12]. ApAn were active against Gram-negative (*E. coli*) and Gram-positive (*S. aureus*) bacteria, but the antimicrobial activity was generally more efficient against Gram-positive *S. aureus* bacteria. This may be due to the composition of the bacterial wall, where, unlike Gram-negative bacteria, Gram-positive bacteria lack a more resistant external membrane [39]. The acylpolyamines isolated from *N. cruentata*, were active against *S. aureus*, *Staphylococcus epidermides*, *Candida albicans* and *Candida glabrata*, however their MIC was not determined [19]. VdTX-I acylpolyamine isolated from the venom of the spider *Vitalius dubius* was evaluated for its antimicrobial activity and also showed activity against a wide spectrum of microorganisms, including the fungus *C. albicans*, in concentrations ranging from 12.5-100 μM [20]. Another acylpolyamine with antimicrobial activity, called mygalin, was isolated from hemocytes of the spider *Acanthoscurria gomesiana*. This molecule, with 417 Da, was tested against *E. coli*, *Micrococcus luteus* and *C. albicans*, but it was active only against *E. coli*, with MIC of 85 µM [17]. The MIC values of ApAn728 (256 µM against *S. aureus*) and of ApAn742 and ApAn756 (128 µM against *S. aureus* and *E. coli*), were relatively higher compared to some antimicrobial peptides, such as the Cupienin 1 peptide, isolated from the venom of the spider *Cupienius salei*. This peptide was active against four bacterial strains, with MIC between 0.08 and 5.0 μM [40]. The peptides isolated from *A. gomesiana* hemocytes, called Gomesina and Acanthoscurrina-1 and -2, also showed important antimicrobial activity. Gomesina peptide was active against Gram-positive bacteria (0.2-12.5 µM), Gram-negative bacteria (0.4-6.25 µM) and fungi (0.4-25 µM) [41], while Acanthoscurrina-1 and -2 peptides were active against *C. albicans* (1.1-2.3 µM) and *E. coli* (2.3-5.6 µM) [42]. On the other hand, higher MIC values are also found for some antimicrobial peptides, similarly to that found for ApAn. For example, the Latarcinas (6a and 7) peptides, isolated from the venom of the spider *Lachesana tarabaevi*, showed antimicrobial activity with MIC greater than 70 µM [43] and the peptide Licotoxin I, isolated from the venom of the spider *Lycosa carolinensis*, presented MIC between 80 and 150 μM against *E. coli* [44]. Despite the high MIC values, the ApAn did not show hemolytic activity against mouse erythrocytes, even in the highest tested concentration (256 µM), differently from what was observed for the VdTXI acylpolyamine of *V. dubius*, which under the concentration of 100 µM showed 6% of hemolysis against human erythrocytes [20]. Regarding mygalin, although no reports have been found on its hemolytic activity, it has been shown that at concentrations between 11.9 and 95.9 µM, mygalin does not interfere with the cell viability of macrophages and splenocytes [45]. Antimicrobial peptides, in turn, can exhibit even more pronounced hemolytic activity, such as Gomesina, which causes 16% of hemolysis in human erythrocytes from low concentrations (1 µM) [41]. Licotoxin I does not exhibit hemolytic activity up to a concentration of 30 µM, but at a concentration of 200 µM, its hemolytic activity on rabbit erythrocytes reaches 55% [44].

## Conclusion

Together, the results of UV spectroscopy and ESI-MS/MS obtained in this work suggested that the acyl aromatic group of acylpolyamines isolated from the *A. natalensis* venom is represented by tyrosine. The identification of this unconventional chromophore for acylpolyamines from spiders demonstrates an even greater diversity of these molecules and that much remains to be discovered. Our results also suggest that the terminal polyamine chain of the ApAn is composed of structural units PA43 or PA53. However, complementary studies using techniques such as nuclear magnetic resonance (NMR) are still necessary for the complete elucidation of the chemical structure of ApAn. In addition, the antimicrobial action against *E. coli* and *S. aureus* and non-hemolytic property of ApAn, may be relevant for the use of these molecules as possible therapeutic agents.

### Abbreviations

ACN: acetonitrile; AMPs: antimicrobial peptides; ApAn: acylpolyamines of *Acanthoscurria natalensis*; ATCC: American Type Culture Collection; CE: collision energy; CXP: collision cell exit potential; DP: decluttering potential; ESI-MS: electrospray ionization mass spectrometry; GS1: ion source gas 1; HCCA: α-cyano-4-hydroxycinnamic acid; MALDI-TOF: matrix associated laser desorption-ionization - time of flight; MH: Mueller-Hinton grown medium; MIC: minimum inhibitory concentration; MRM: multiple reaction monitoring; ROS: reactive oxygen species; RP-HPLC: reverse-phase liquid chromatography; TFA: trifluoroacetic acid; UV: ultraviolet spectroscopy.

## Supplementary material

The following online material is available for this article:

Additional file 1. ESI-MS and MS/MS spectra of ApAn728. **(A)** The protonated ion [M+H]+ at m/z 729 was detected in MS mode. **(B)** Fragmentation spectrum MS/MS of ion at m/z 365.

Additional file 2. ESI-MS spectrum of ApAn614a. The protonated ion [M+H]+ at m/z 615 was detected in MS mode.

Additional file 3. ESI-MS spectrum of ApAn614b. The protonated ion [M+H]+ at m/z 615 was detected in MS mode.

Additional file 4. ESI-MS and MS/MS spectra of ApAn742. **(A)** The protonated ion [M+H]+ at m/z 743 was detected in MS mode. **(B)** Fragmentation spectrum MS/MS of ion at m/z 372.

Additional file 5. ESI-MS and MS/MS spectra of ApAn756. **(A)** The protonated ion [M+H]+ at m/z 757 was detected in MS mode. **(B)** Fragmentation spectrum MS/MS of ion at m/z 379.
