# Supplementary material for: Antimicrobial activity and partial chemical structure of acylpolyamines isolated from the venom of the spider Acanthoscurria natalensis
Source: J Venom Anim Toxins Incl Trop Dis. 2022 Mar 18;28:e20210017. doi: 10.1590/1678-9199-JVATITD-2021-0017 (PMC8939072; doi:10.1590/1678-9199-JVATITD-2021-0017)
Supplement: Additional file 4. [file 1678-9199-jvatitd-28-e20210017-s4.pdf]

J Venom Anim Toxins incl Trop Dis
https://doi.org/10.1590/1678-9199-JVATITD-2021-0017

Supplementary Material to "Antimicrobial activity and partial chemical structure of acylpolyamines isolated from the venom of the spider *Acanthoscurria natalensis*"

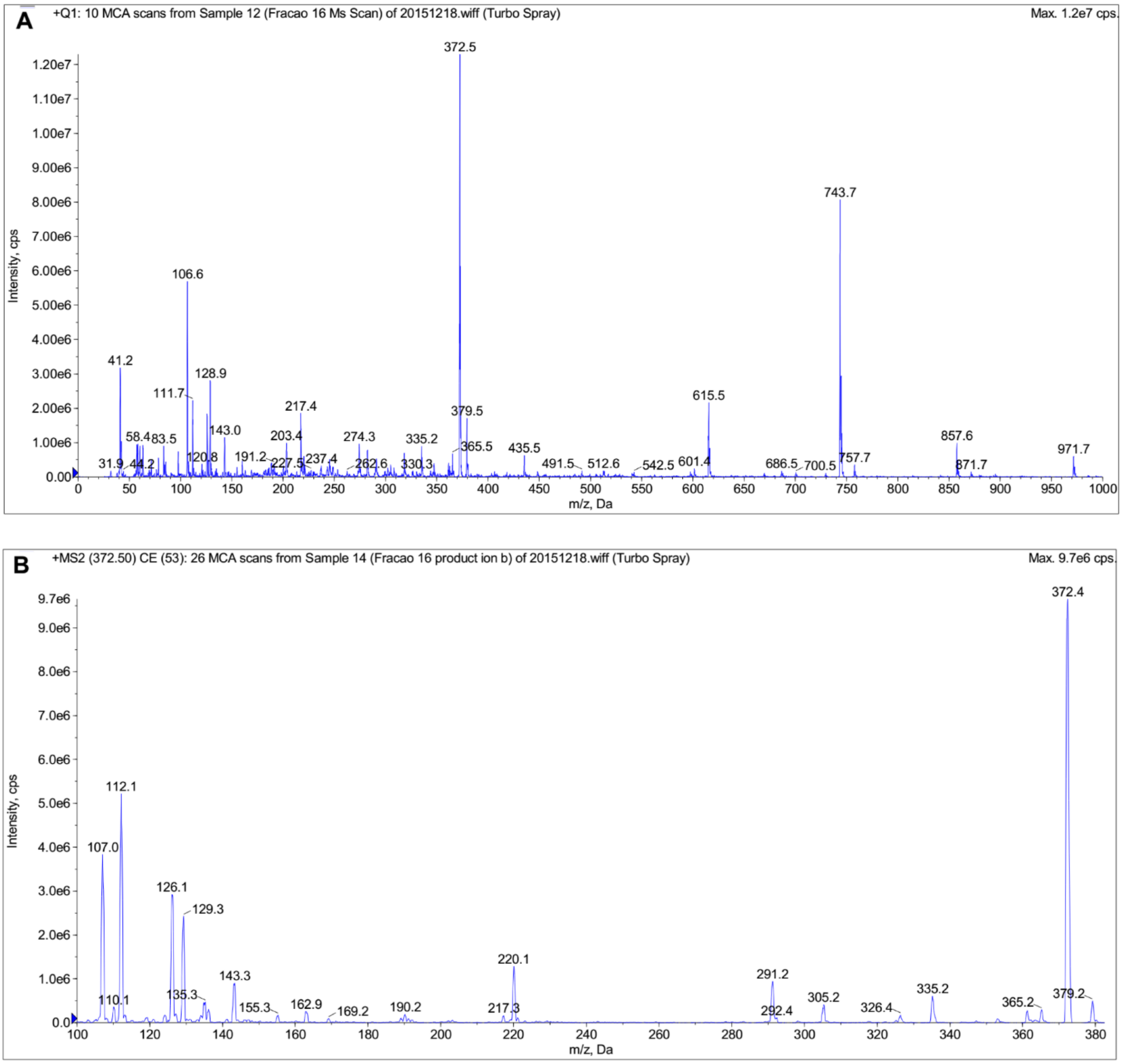

Additional file 4. ESI-MS and MS/MS spectra of ApAn742. **(A)** The protonated ion [M+H]+ at m/z 743 was detected in MS mode. **(B)** Fragmentation spectrum MS/MS of ion at m/z 372.
